# The Nanostructure of Polymer-Active Principle Microparticles Produced by Supercritical CO_2_ Assisted Processing

**DOI:** 10.3390/nano12091401

**Published:** 2022-04-19

**Authors:** Ernesto Reverchon, Mariarosa Scognamiglio, Lucia Baldino

**Affiliations:** Department of Industrial Engineering, University of Salerno, Via Giovanni Paolo II 132, 84084 Fisciano, SA, Italy; ereverchon@unisa.it (E.R.); mrscogna@unisa.it (M.S.)

**Keywords:** biopolymer microparticles, drug nanoparticles, nanostructure, drug delivery, supercritical CO_2_ processing

## Abstract

Traditional and supercritical CO_2_ assisted processes are frequently used to produce microparticles formed by a biopolymer containing an active principle to improve the bioavailability of the active principle. However, information about the internal organization of these microparticles is still scarce. In this work, a suspension of dextran + Fe_3_O_4_ nanoparticles (model system) and a solution of polyvinylpyrrolidone (PVP) + curcumin were used to produce spherical microparticles by supercritical CO_2_ processing. Periodic dynamic light scattering measurements were used to analyze the evolution of the microparticles dissolution, size, and size distribution of the guest active principle in the polymeric matrix. It was found that curcumin was dispersed in the form of nanoparticles in the PVP microparticles, whose size largely depended on its relative concentration. These results were validated by transmission electron microscopy and scanning electron microscopy of the PVP microparticles and curcumin nanoparticles, before and after the dissolution tests.

## 1. Introduction

Many active pharmaceutical ingredients (APIs) require an improvement of their dissolution kinetics to obtain a good therapeutic performance and to optimize their efficiency [1]. One of the techniques used to obtain APIs fast/controlled dissolution rates is the reduction of the particle size. Indeed, it is well known that, in the case of compounds characterized by a poor solubility in water, a reduction of particle size accelerates their dissolution kinetics and increases their bioavailability [1,2]. Therefore, the production of micro-sized APIs is frequently attempted in scientific literature [2,3,4] and in industrial practice. A further improvement of the dissolution kinetics can be obtained when API nanoparticles are produced. However, nanoparticles are very difficult to obtain, and their handling is complex due to problems of collection and the flowability of these materials [5,6].

A possible solution is to produce carrier-API microparticles, where the carrier is, as a rule, a biocompatible polymer, and the API is dispersed in it, possibly at nanometric scale. The traditional processes frequently used to produce biopolymeric microparticles are: spray drying [7,8], freeze drying [9,10], solvent evaporation [11,12], coacervation [13,14], ionic gelation [15,16], interfacial polymerization [17,18], and molecular inclusion complexation [19,20]. However, these methods show numerous drawbacks, such as: (i) difficulty in controlling particle size and shape, (ii) broad particle size distribution, (iii) relatively high drying temperatures that can damage biomolecules, (iv) low production yield, (v) long processing time, (vi) high capital and operating costs, (vii) low drug encapsulation efficiency, (viii) post-processing steps to remove monomers, by-products, organic solvent residues and surfactants, and (ix) poor physical and chemical stability of the products over time [18,19,20].

To overcome at least in part these problems, supercritical CO_2_ (SC-CO_2_) assisted processes have been proposed to obtain carrier-API microparticles. Among the others, supercritical assisted atomization (SAA) [21,22], supercritical antisolvent precipitation (SAS) [23,24,25], supercritical emulsion extraction (SEE) [26,27], and supercritical assisted electrospray [28,29], are the most frequently used. Thanks to the properties of CO_2_ at supercritical conditions [30], such as a gas-like diffusivity, near zero surface tension, and liquid-like density, it is possible to perform faster processes with respect to the traditional ones that assure [31,32,33]: (i) complete organic solvent removal, (ii) controlled shape and narrow size distribution of the microparticles, (iii) high drug encapsulation efficiency, and (iv) that the mild operative conditions generally selected for these processes are suitable to treat thermolabile biomolecules. In particular, during SAA processing, a gas expanded liquid (GXL) is first created in a saturator, due to the dissolution of SC-CO_2_ in the polymer + API solution and then the expanded liquid solution is sprayed through an injection nozzle in a precipitation chamber. After droplets drying obtained by hot nitrogen, the corresponding microparticles are formed and collected on a stainless steel filter [22].

Some attempts have been previously performed to describe the structure of the microspheres obtained by SC-CO_2_ assisted processes. Adami and Reverchon [22] produced dextran and chitosan microparticles by SAA, loaded with magnetite nanoparticles. These authors used SEM (scanning electron microscope), EDX (energy dispersive X-ray), and TGA (thermogravimetric analysis) to obtain information on morphology, particle size and size distribution, and loading of the nanoparticles in the polymeric matrix. In another work, curcumin was loaded in polyvinylpyrrolidone (PVP) microparticles, produced by SAA, in order to enhance the drug bioavailability [34]. Physico-chemical characterizations demonstrated that the microspheres were amorphous and curcumin was intimately mixed with the polymer. Moreover, UV-Vis spectrophotometric analyses confirmed a drug encapsulation efficiency between 94 and 100%; whereas dissolution tests showed that curcumin was released up to 4.5 times faster with respect to the physical mixture. Di Capua et al. [35] increased the β-carotene dissolution rate up to 22 times when it was co-precipitated in PVP microspheres by SAA.

However, only indirect information about microparticles’ nanostructure was collected in these works. For example, drug release tests are generally used to show the faster dissolution of poorly-water soluble drugs encapsulated in microparticles with respect to the untreated API [24,28,34,36,37]. The possess of the nanostructural information instead could be relevant to better understand the precipitation mechanisms involved and their influence on the nanostructural organization of the microparticles. For example, core-shell nanoparticles dispersion [38] or molecular dispersion [39] could be some of the possible particle organizations. From a practical point of view, nanostructural information can help the formulation of co-precipitates.

A technique that can be used to obtain direct nanostructural information about the microparticles internal organization is transmission electron microscopy (TEM) that allows to observe the section and size of microparticles and their content. However, a limited number of particles can be observed and their size distribution can be only argued. Moreover, samples preparation can be very complex [40]. In some cases, dynamic light scattering (DLS) analysis has been proposed in the literature to study the size evolution of nano/microparticles over time [40,41,42]. For example, Lyutova et al. [42] demonstrated by DLS that arginine shifted the population of nanoparticles with higher hydrodynamic radii to the lower ones, suggesting that arginine reduced the protein aggregation process thanks to the suppression of intermolecular interactions among aggregation-prone molecules.

To summarize, the analysis of the literature suggests the relevance of obtaining further information about microparticles nanostructure, which is useful for understanding their internal organization and, consequently, their formation mechanisms.

In this work, for the first time, DLS periodic measurements were systematically used to follow over time the dissolution of the polymeric part of carrier-API microparticles, trying to evidence size and size distribution of the active compound contained in it. To obtain this result, polymers and active materials, characterized by different dissolution behaviors in a given organic solvent, were selected; i.e., active materials should be practically not soluble or poorly-soluble in the solvent used for DLS analysis. With this aim, composite microparticles of dextran-Fe_3_O_4_ produced by SAA, in which Fe_3_O_4_ nanoparticles diameter was known, were used as a test material to set up the method. Then, microparticles of PVP-curcumin, obtained using the same process, were tested to measure size and size distribution of the curcumin nanoparticles dispersed in the biopolymeric structure. DLS results were compared with TEM and SEM analysis.

## 2. Materials and Methods

**Figure 1:**
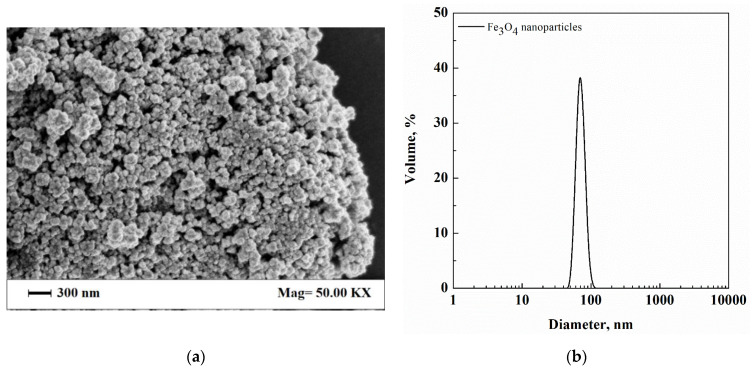
(**a**) SEM image of Fe_3_O_4_ nanoparticles and (**b**) Fe_3_O_4_ nanoparticles size distribution measured by DLS.

Dextran from *Leuconostoc mesenteroides* (Dextran 40, average M_w_ 35,000–43,000 g/mol), magnetite (Fe_3_O_4_) nanopowder (>98% trace metal basis), and Tween^®^ 80 (M_w_ 1310 g/mol) were supplied by Sigma Aldrich (Milan, Italy) and were used to prepare the first composite system. In particular, Fe_3_O_4_ nanoparticles showed a mean diameter of about 70 nm, as observed by SEM (Figure 1a) and measured by DLS (Figure 1b).

Curcumin (Cur, 99% purity, Sigma Aldrich, Milan, Italy), polyvinylpyrrolidone (PVP, M_w_ 10,000 g/mol, Fluka, Milan, Italy) and ethanol (99.5% purity, Sigma Aldrich, Milan, Italy) were used to prepare the second microparticulate system.

Distilled water was produced in laboratory, using a homemade lab-scale distiller. Nitrogen (N_2_, 99% purity, SOL, Milan, Italy) and carbon dioxide (CO_2_, 99.9% purity, Morlando Group, Naples, Italy) were used to carry out SAA processing.

### 2.1. Microparticles Preparation Procedure and SAA Plant Description

Dextran-Fe_3_O_4_ suspensions were prepared dissolving the polymer (200 mg/mL solution concentration) in water and adding Tween^®^ 80 7.5% *w*/*w*; then, magnetite nanoparticles, at 10% *w*/*w* with respect to the polymer, were added and the system was sonicated using a high-intensity ultrasonic probe (mod. S-450D, Branson Ultrasonic Corp. Danbury, CT, USA) for 1 min, operating at 50% amplitude, using the pulse mode [22].

PVP was dissolved in ethanol (10 mg/mL solution concentration); then, curcumin was added at different Cur-PVP weight ratios (1/2, 1/4, 1/6, 1/8) [34]. The system was stirred at 100 rpm and at room temperature, until a homogeneous solution was formed.

The SAA apparatus consisted of two high-pressure pumps (mod. 305, Gilson, Cinisello Balsamo (MI), Italy) to deliver the polymeric suspension or solution and liquid CO_2_ to a heated saturator. The saturator was a high-pressure vessel (50 cm^3^ internal volume) loaded with stainless steel perforated saddles, used to allow a large contact surface between the liquid suspension or solution and CO_2_, to form a GXL. GXL was sprayed through a nozzle (80 μm internal diameter) into the precipitation vessel (3 dm^3^ internal volume), operated at atmospheric pressure. A controlled flow of N_2_, previously heated using an electric heat exchanger (mod. CBEN 24G6, Watlow, Corsico (MI) Italy), was sent to the precipitator to induce drying of the droplets. A stainless steel filter, located at the bottom of this chamber, was used to collect the dried particles, while the gaseous stream flowed out. The apparatus was completed by a separator for the recovery of the liquid solvent. SAA layout and further details about the experimental procedures are described in [22,34].

### 2.2. Characterization Methods of the Microparticles

The morphology of the microparticles was observed by a field emission scanning electron microscope (FE-SEM, mod. LEO 1525, Carl Zeiss SMT AG, Oberkochen, Germany). Powder samples were dispersed on a carbon tab, previously stuck to an aluminum stub (Agar Scientific, Stansted, UK), and coated with gold using a sputter coater (mod. 108 Å, Agar Auto Sputter Coater, Stansted, UK) at 40 mA for 120 s.

Particle size (PS) and particle size distribution (PSD) were measured by a dynamic light scattering (DLS) (mod. Zetasizer Nano S, Worcestershire, UK). The same instrument was used for the periodic DLS measurements, obtained by the repetition, at fixed time intervals (every 5 min up to microparticles complete dissolution), of the DLS analysis on the same sample. The polymer (dextran or PVP) was soluble in the liquid medium used to perform DLS; whereas the other compound (Fe_3_O_4_ or Cur) was not soluble in it. Operating in this way, consecutive measurements can indicate the progressive dissolution of the polymer and the release of the co-precipitated material. In particular, 1 mg of each microparticulate systems was firstly tested by DLS using 2 mL (0.5 mg/mL microparticles concentration) of ethyl acetate as a liquid medium, since all the compounds are not soluble in it, to obtain the mean diameter of the starting microparticles. Then, periodic dissolution tests were carried out by DLS using the same concentration microparticles in the liquid medium, which was water for dextran-Fe_3_O_4_ microparticles and phosphate buffered saline (PBS) at pH 7.4 for Cur-PVP microparticles. In this last case, it is worth to note that ethanol cannot be used as the dissolution medium since both PVP and curcumin are soluble in it. Therefore, PBS at pH 7.4 was selected, taking into account that the curcumin solubility in this liquid medium is about 1.60 µg/mL [43] and that, in all of the cases tested, the amount of curcumin released from 1 mg of PVP microparticles in 2 mL of PBS largely exceeded this solubility limit. Operating in this way, it was possible to measure the curcumin size and size distribution since it was dispersed in the liquid medium. All the analyses were performed in triplicate.

Transmission electron micrographs (TEM) were obtained using a cryo-TEM (TECNAI by FEI, Hillsboro, OR, USA). A sample volume of 20 µL was dropped on Formvar/Carbon membranes (Agar Scientific, Stansted, UK); after that, they were dried overnight and at room temperature before the analysis.

## 3. Results and Discussion

### 3.1. Dextran-Fe_3_O_4_ Composite Microparticles: Model System

The first system tested in this work was formed by dextran-Fe_3_O_4_ composite microparticles. This is a model system since Fe_3_O_4_ nanoparticles formed a suspension in the dextran aqueous solution, and their characteristics were known in advance, as shown in Figure 1a,b.

**Figure 2:**
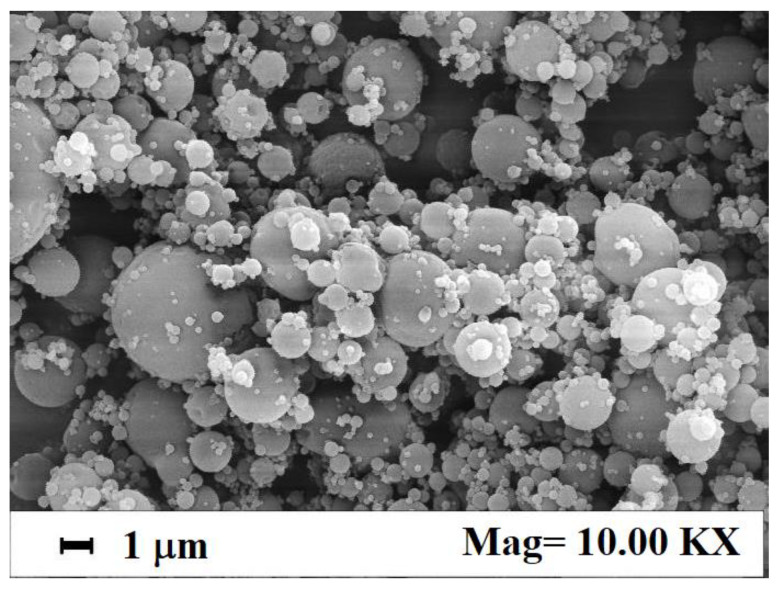
SEM image of dextran microparticles loaded with 10% *w*/*w* Fe_3_O_4_ nanoparticles, produced by SAA in this work.

These composite microparticles were successfully produced in a previous study using SAA [22]. In that work, operating at 95 bar and 85 °C in the saturator, and setting a gas-to-liquid ratio (GLR) at 1.8, different loadings of Fe_3_O_4_ nanoparticles (i.e., 10%, 20% and 30% *w*/*w*) in the starting solution were tested. A maximum nanoparticles encapsulation efficiency of about 72% was obtained [22]. Some of those experiments were replicated in this work, using the same operative conditions for SAA process, and a Fe_3_O_4_ nanoparticles loading of 10% *w*/*w* in the starting dextran solution. Figure 2 shows a SEM image of these newly produced composite microparticles and confirms their spherical and well-defined shape, with a mean size lower than 2 µm.

**Figure 3:**
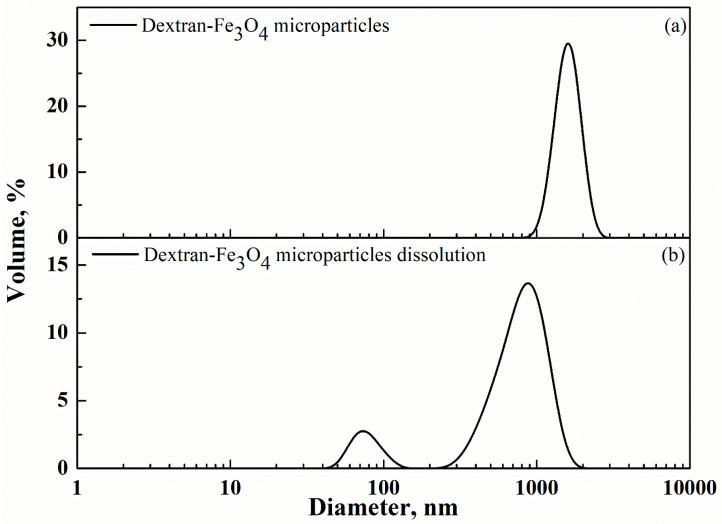
DLS traces of: (**a**) starting dextran-Fe_3_O_4_ microparticles and (**b**) intermediate dextran-Fe_3_O_4_ microparticles dissolution.

Microparticles loaded with 10% *w*/*w* Fe_3_O_4_ were tested by DLS in ethyl acetate (in which both dextran and Fe_3_O_4_ nanoparticles are not soluble) to have an indication of their starting mean size. A particle mean diameter of 1.61 µm and a standard deviation of 0.28 µm were measured (Figure 3a). Then, periodic DLS was carried out on the composite microparticles in water, in which only dextran was soluble. The result obtained is summarized in Figure 3b. It shows that the composite microparticles started to dissolve and their PS progressively decreased; meanwhile, a DLS peak, characterized by a mean diameter of 81 ± 21 nm, appeared, which corresponded to the progressive release of Fe_3_O_4_ nanoparticles. At the end of the dissolution process (after 15 min), only one peak was indicated by DLS and overlapped with the one in Figure 1b: i.e., it was related to the complete release of Fe_3_O_4_ nanoparticles in the dissolution medium. It is worth to note that the volume% of dextran-Fe_3_O_4_ microparticles also reduced during the dissolution test (compare the *y*-axis of Figure 3a,b) and the PSD showed an enlargement due to the simultaneous presence of microparticles at different dissolution stages. Also, the DLS peak related to Fe_3_O_4_ nanoparticles was somewhat enlarged during the dissolution process, suggesting that very small quantities of dextran still covered Fe_3_O_4_ native nanoparticles during the last minutes of the dissolution process.

### 3.2. PVP-Curcumin Microparticles

Encouraged by the results obtained for the model system, in the following part of the work, periodic DLS measurements were used to study the dissolution of Cur-PVP microparticles. This system is more complex than the previous one, since, in this case, both PVP and curcumin solubilized in ethanol (see Section 2); therefore, the mean size of the curcumin particles embedded in the polymeric matrix after processing was not previously known and, changing the relative proportion of the two compounds, curcumin particles of different diameters could be formed.

As discussed in the Introduction, Cur-PVP microparticles were produced by SAA in a previous work of our research group [34], working at 95 bar and 80 °C in the saturator, and using a 1.8 GLR. In that work, spherical microparticles were obtained, and the release tests, carried out on the microparticles at different Cur-PVP weight ratios, demonstrated an increase of curcumin dissolution rate up to about 4.5 times with respect to the untreated curcumin powder [34].

**Figure 4:**
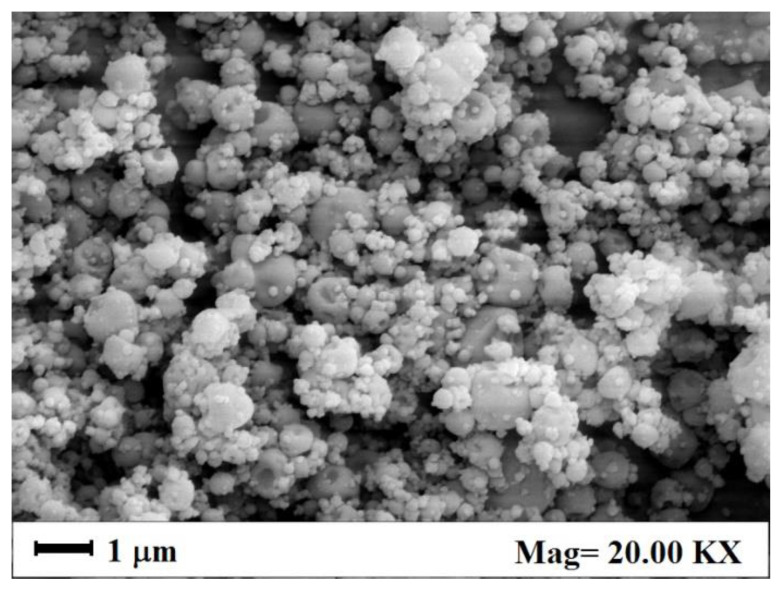
SEM image of Cur-PVP microparticles produced by SAA in this work, at a Cur-PVP weight ratio of 1/8.

These results in the literature were reproduced in a new set of experiments carried out in this study. As an example, a SEM image of Cur-PVP microparticles, obtained by SAA at the operative conditions previously described, and at a Cur-PVP weight ratio of 1/8, is reported in Figure 4. It shows spherical and not coalescing Cur-PVP microparticles.

**Figure 5:**
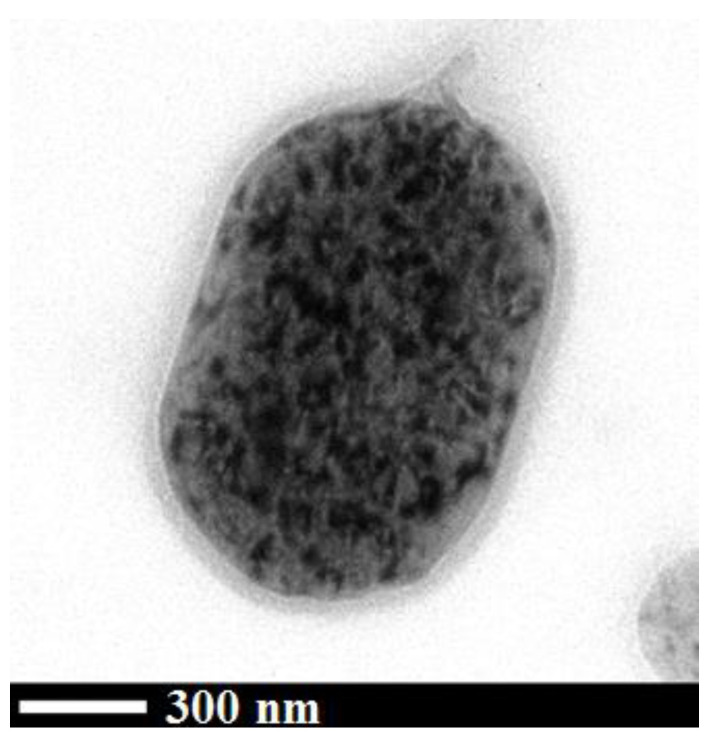
TEM image of 1/8 Cur-PVP sample.

In this case, a TEM analysis of the samples was also performed to have an indication of the structure of curcumin dispersed in the polymeric matrix. The TEM image in Figure 5 is related to the 1/8 Cur-PVP sample and shows that curcumin was dispersed in the form of nanoparticles (darker dots in the image) in the PVP matrix; but their diameter was very difficult to measure since many nanoparticles were simultaneously present on different levels in the PVP microparticles. Therefore, in this case, the TEM analysis has given only a semi-qualitative indication of the microparticle internal structure, evidencing that curcumin was organized in nanometric aggregates. As reported in the Introduction, this evidence confirms that the co-precipitated compound (curcumin) was not dispersed at a molecular level in the polymeric structure and that a nucleation process followed by a reduced growth of the active compound has characterized the condensation of SAA droplets.

**Figure 6:**
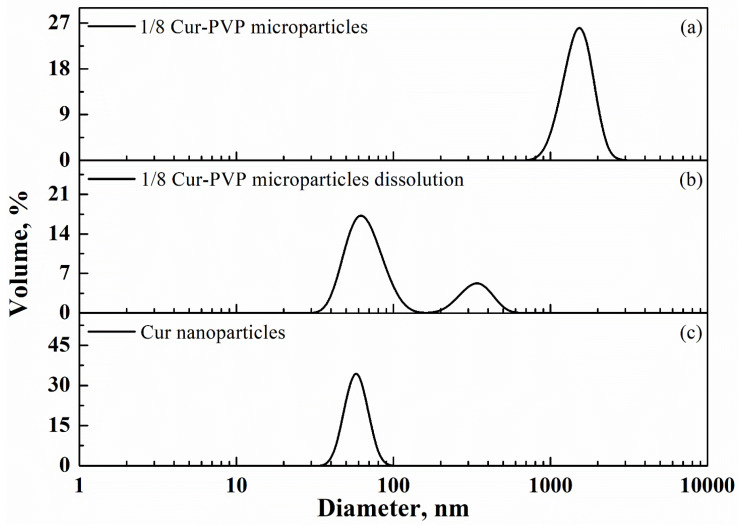
DLS traces of: (**a**) starting 1/8 Cur-PVP microparticles, (**b**) 1/8 Cur-PVP microparticles dissolution, (**c**) final Cur nanoparticles. The samples were tested every 5 min, and only the most significant results are reported in this figure.

At this point of the work, periodic DLS measurements were systematically carried out on powder samples obtained at Cur-PVP ratios of 1/2, 1/4, 1/6, and 1/8 by weight. For example, for the 1/8 Cur-PVP microparticles, only one peak was observed at the beginning of the analysis, with a 1.52 µm mean diameter (0.30 µm standard deviation), as illustrated in Figure 6a (test performed in ethyl acetate in which PVP and curcumin are not soluble). When PBS at pH equal to 7.4 was used, in which only PVP was soluble, the DLS trace evolved as in Figure 6b, i.e., two peaks appeared: the first one, on the right, represented the dissolving PVP microparticles (0.34 ± 0.07 µm vs. 1.52 ± 0.30 µm diameter), characterized by a smaller diameter and peak volume with respect to Figure 6a (5% vs. 26%), and a second peak, related to a nanometric material, was also shown. This second peak, on the left of the trace, represented curcumin nanoparticles that were released in the liquid medium; their mean diameter was 68 ± 17 nm. Figure 6c shows the end of the dissolution process: only the peak related to curcumin nanoparticles was present and the final mean diameter was 66 ± 10 nm. It is worth noting that the peak in Figure 6c is sharper than those observed during the evolution of the dissolution process. It means that small aggregates of Cur-PVP were formed during the dissolution process and/or PVP residues surrounded curcumin particles until the polymer was definitively dissolved in the liquid medium, at the end of the analysis.

The same observations were produced for 1/2, 1/4, and 1/6 Cur-PVP weight ratios. The corresponding periodic DLS traces are not reported for the sake of synthesis; but they are consistent with the previous results.

Therefore, periodic DLS measurements, performed in the opportune dissolution medium, allow to obtain initial microparticles size distribution and final curcumin nanoparticles size distribution. Moreover, they can give an indication about the evolution of the dissolution process; i.e., microparticles showed a progressive size reduction, due to PVP solubilization, and a second PSD peak appeared at a nanometric level that was related to curcumin nanoparticles contained in the starting microparticles.

**Figure 7:**
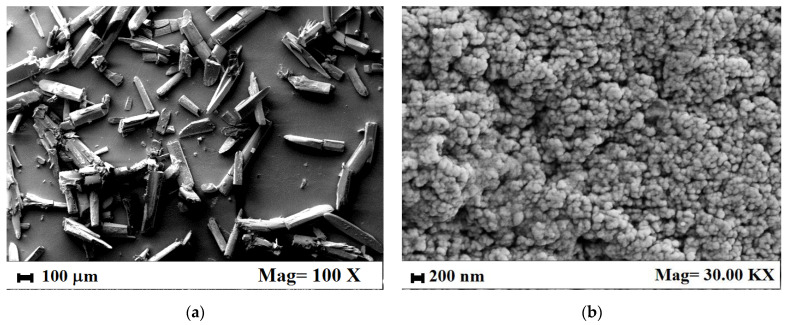
SEM images of (**a**) untreated curcumin powder and (**b**) curcumin nanoparticles collected after DLS dissolution analysis.

In Figure 7, two SEM images, related to untreated curcumin powder (Figure 7a) and curcumin nanopowder collected at the end of the DLS periodic measurement (Figure 7b), are reported. Untreated curcumin powder showed a needle-like geometry and was crystalline [44]. Curcumin nanoparticles, collected after PVP microparticles dissolution in PBS, instead, were irregularly spherical, amorphous, and showed a mean diameter lower than 100 nm, in line with DLS results, i.e., they qualitatively confirm the DLS observations about the nanometric dimension of curcumin contained in the PVP microparticles. SEM analysis also confirms the results of X-ray powder diffractometer and differential scanning calorimetry previously discussed in [34]. Therefore, the hypothesis that curcumin was homogeneously dispersed at a nanometric level in the PVP microparticles produced by SAA has been definitively demonstrated in this work.

The initial microparticles mean diameter and final curcumin nanoparticles mean diameter are summarized in Table 1, covering all the Cur-PVP ratios analyzed in this work.

**Figure 8:**
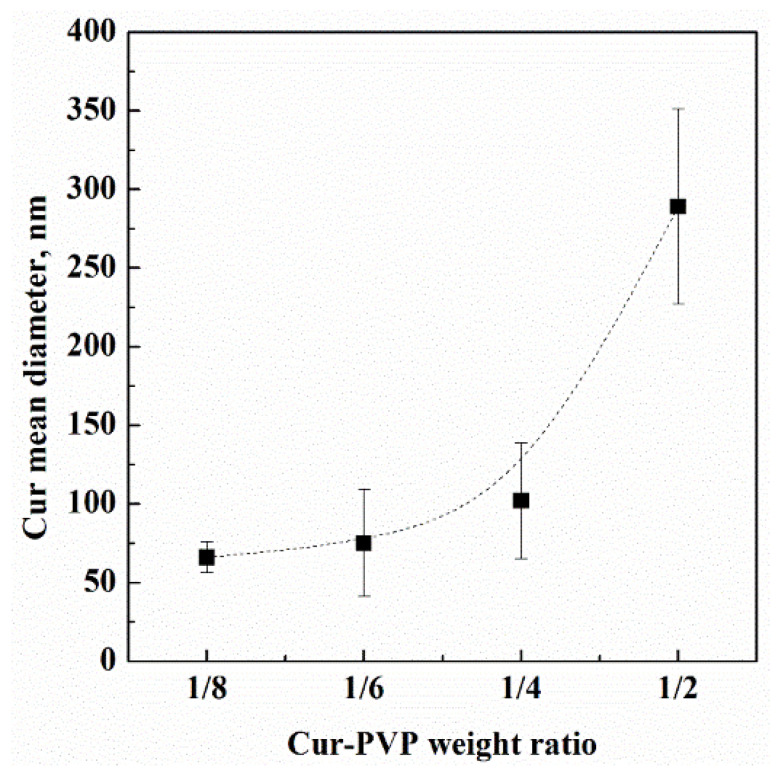
Curcumin mean diameter evolution (highlighted by the dashed line) in PVP microparticles, depending on the Cur-PVP weight ratio.

The data in Table 1 show that by increasing the Cur-PVP weight ratio from 1/8 to 1/2 (i.e., increasing the relative curcumin concentration in the starting formulation), the diameter of the corresponding curcumin nanoparticles increased, indicating that nucleation and growth process of curcumin inside the PVP-based microparticles depended on the initial curcumin concentration, and the growth of nanoparticles was favored at higher curcumin concentrations. It is possible to propose a diagram showing the trend observed for curcumin nanoparticles diameter, as reported in Figure 8 (the vertical bar is the standard deviation); this trend is not linear at the highest Cur-PVP ratio.

These observations can be correlated with the drug release tests performed in the previous literature [34]. In particular, those results showed that the lower the Cur-PVP ratio (i.e., the smaller the curcumin quantity contained in the starting solution was), the faster its release. Therefore, assuming that the dissolution rate of PVP in PBS at pH 7.4 is in all cases the same, independently of the content of the guest molecule, when the smaller curcumin nanoparticles are present in the PVP microparticles, their dissolution rate is faster.

## 4. Conclusions

The Dextran-Fe_3_O_4_ model system allowed us to validate the periodic DLS analysis as a method to obtain information about the internal microparticles nanostructure. Applying this method to Cur-PVP microparticles, the size and size distribution of curcumin nanoparticles were successfully measured, completing the TEM indications, i.e., during the supercritical CO_2_ processing, curcumin nanoparticles were formed inside the polymeric matrix. Nanoparticles mean diameter measured by DLS was also approximatively confirmed by SEM analysis of curcumin powder after PVP dissolution tests.

In the next future, periodic DLS method can be extended to the analysis of other composite systems to search for possible different internal organizations of the produced microparticles.

## Figures and Tables

**Table 1:** Mean diameters measured by DLS of Cur-PVP microparticles and curcumin nanoparticles, tested in this work.

Cur-PVP Ratio by Weight	Microparticles Mean Diameter ± Standard Deviation, nm	Cur Nanoparticles Mean Diameter ± Standard Deviation, nm
1/2	2530 ± 440	289 ± 62
1/4	2180 ± 580	102 ± 37
1/6	1790 ± 270	75 ± 34
1/8	1520 ± 300	66 ± 10

## Data Availability

Not applicable.
